# From episodic to habitual prospective memory: ERP-evidence for a linear transition

**DOI:** 10.3389/fnhum.2014.00489

**Published:** 2014-07-02

**Authors:** Beat Meier, Sibylle Matter, Brigitta Baumann, Stefan Walter, Thomas Koenig

**Affiliations:** ^1^Institute of Psychology, Experimental Psychology and Neuropsychology, University of BernBern, Switzerland; ^2^Center for Cognition, Learning and Memory, University of BernBern, Switzerland; ^3^Department of Psychiatric Neurophysiology, University Hospital of PsychiatryBern, Switzerland

**Keywords:** intention, habit, recognition, covariance mapping, N300, P3b, parietal old/new effect, prospective positivity

## Abstract

Performing a prospective memory task repeatedly changes the nature of the task from episodic to habitual. The goal of the present study was to investigate the neural basis of this transition. In two experiments, we contrasted event-related potentials (ERPs) evoked by correct responses to prospective memory targets in the first, more episodic part of the experiment with those of the second, more habitual part of the experiment. Specifically, we tested whether the early, middle, or late ERP-components, which are thought to reflect cue detection, retrieval of the intention, and post-retrieval processes, respectively, would be changed by routinely performing the prospective memory task. The results showed a differential ERP effect in the middle time window (450–650 ms post-stimulus). Source localization using low resolution brain electromagnetic tomography analysis suggests that the transition was accompanied by an increase of activation in the posterior parietal and occipital cortex. These findings indicate that habitual prospective memory involves retrieval processes guided more strongly by parietal brain structures. In brief, the study demonstrates that episodic and habitual prospective memory tasks recruit different brain areas.

## INTRODUCTION

Typically, habits are formed without intention. However, there are situations in which we intentionally and deliberately want to form a habit, for example, when we must remember to take medication on a regular basis. This situation is referred to as habitual prospective memory and the neural basis of its formation is the goal of the present study. Prospective memory can be defined as the ability to remember to perform a previously formed intention at the appropriate occasion. It is highly relevant in everyday life and is involved in tasks such as remembering to buy groceries on the way home from work, to keep an appointment or to comply with a medication prescription regiment. Prospective memory tasks can be classified as episodic when they are concerned with one-time events and they can be classified as habitual when they need to be executed repeatedly (cf., Meacham and Singer, 1977; Meacham and Leiman, 1982; Kvavilashvili and Ellis, 1996; Einstein et al., 1998; Graf, 2005). Although there has been a considerable interest in prospective memory in the last two decades, the main focus was on episodic prospective memory tasks. In particular, the question whether remembering an episodic prospective memory task can occur spontaneously or whether strategic monitoring for the retrieval occasion is necessary is at the core of the current theoretical debate (see McDaniel and Einstein, 2007; Kliegel et al., 2008 for overviews).Only a few studies were concerned with habitual prospective memory (Einstein et al., 1998; Elvevag et al., 2003; Vedhara et al., 2004; Matter and Meier, 2008; Cuttler and Graf, 2009) and these studies were mainly concerned with habitual prospective memory performance deficits in older adults and in patient populations, or with questions related to medication adherence. However, none of these studies has examined the neural correlates of habitual prospective memory. The goal of the current study was to fill this gap and to identify the electrophysiological signature of the transition from episodic to habitual prospective memory using event-related potentials (ERPs).

In previous ERP studies different characteristic modulations of prospective memory have been identified. The *N300* represents an occipital–parietal negativity in an early time window about 300 ms after stimulus-onset and is elicited when prospective targets are compared to ongoing task trials, or when remembered targets are compared to missed prospective memory target trials (West and Covell, 2001; West et al., 2001; West and Ross-Munroe, 2002; West, 2005, 2008). Moreover, the N300 is sensitive to the amount of available attentional resources, that is, increased attentional demands of the ongoing task disrupted the efficiency of prospective memory target detection and led to an attenuation of the N300. Therefore, this component is associated with processes related to the detection of the prospective memory targets and can be considered as the prospective component of a prospective memory task (i.e., remembering that something must be done).

The prospective positivity occurs between 400 and 1200 ms after stimulus-onset which is distributed across central, parietal, and occipital brain areas (see West, 2005, 2008). This positivity is elicited when prospective memory target trials are compared to prospective lures and also, when prospective memory target trials are compared to ongoing task trials (West et al., 2001; West and Ross-Munroe, 2002; West and Krompinger, 2005). This component can be further subdivided into three components, Pb3, parietal old/new effect, and sustained parietal positivity (West, 2011). The *P3b* is a relatively large positivity over parietal regions and it typically peaks between 300 and 800 ms post-stimulus. It is elicited when infrequent targets are detected, for example during the oddball task (e.g., Kok, 2001). A further component is the *parietal old/new effect*, an effect typically found in studies of recognition memory (Rugg and Curran, 2007). Therefore, it is thought to be associated with processes related to the retrieval of the intention and can be considered as the retrospective component of a prospective memory task (i.e., remembering what has to be done). Both the *P3b* and the *parietal old/new effect* occur in about the same time window, but can be distinguished by their functional relevance.

In addition, a further component which occurs in the later part of this time window and which is expressed mainly on parietal electrodes has been identified. This *sustained parietal positivity* is thought to be related to post-retrieval processes which may support the realization of the intention once it is retrieved (West and Krompinger, 2005; West, 2007; West et al., 2007). Thus, the prospective component (remember that) and the retrospective component (remember what), which are inherent in a prospective memory task, are supported by different ERP-components (Zimmermann and Meier, 2006, 2010; West et al., 2007).

So far, it is not known whether the ERP-components are differentially associated with episodic and habitual prospective memory. It has been proposed that as a task becomes habitual, it requires less attention and its execution becomes more automatic (Einstein et al., 1998; Dismukes, 2008). Therefore it is possible that the detection of prospective memory targets requires less attention and as a consequence, the N300 which has been shown to depend on attentional processes may be attenuated as a task becomes habitual. Moreover, studies with the oddball paradigm have shown that with habituation the P3b is reduced and thus a reduction of the P3b might also be expected when a prospective memory task becomes habitual (Ravden and Polich, 1998).

However, when the dual-task nature of a prospective memory task is considered the opposite result is also possible (cf., Smith, 2003; Bisiacchi et al., 2009). In dual-task paradigms the P3b produced by a secondary task typically decreases in amplitude when the difficulty of a primary task is increased (Strayer and Kramer, 1990; Kramer et al., 1991; Watter et al., 2001). In a habitual prospective memory task the difficulty of the primary (i.e., ongoing) task typically decreases with practice. Thus, the amplitude of the secondary (i.e., the prospective memory task) may increase when the task changes from episodic to habitual. Moreover, with increasing practice of responding to the prospective memory target events this particular stimulus category may be integrated into the task-set of the ongoing task, thus leading to a change of the dual-task structure, and as a consequence to a change in resource allocation (i.e., an increase in spontaneous retrieval).

Further, it is also possible, that by repeatedly retrieving a particular task the parietal old/new effect is affected. From studies of recognition memory it is known that this component is enhanced when confidence in recollection is increased (Curran, 2004). Moreover, as a task becomes habitual the execution of the intention may also become more automatized and accordingly a change in the sustained parietal positivity may occur.

To test these possibilities we conducted two experiments. In Experiment 1, we used verbal materials. The ongoing task was a lexical decision task and the prospective memory task was to respond to a specific target word. In Experiment 2, we used non-verbal materials. The ongoing task was a perceptual discrimination task using abstract shapes and the prospective memory task was to respond to the category of white shapes. In both experiments, a total of forty prospective memory targets occurred and the main question was whether we would find differences between ERP components of the first versus the second half of the experiment.

In order to investigate the neural signature of the episodic to habitual transition, we defined three time windows which were derived from previous ERP studies: an early time window lasting from 250 to 450 ms after stimulus-onset to assess the N300, a middle time window lasting from 450 to 650 ms after stimulus-onset to assess the P3b and the parietal old/new effect and a late time window lasting from 650 to 850 ms to assess the sustained parietal positivity.

## EXPERIMENT 1

### METHOD

#### Participants

Twenty-two right-handed psychology students (mean age = 26.5 years, SD = 8.1; 16 female) participated in the study. They were recruited from the departmental subject pool and received course credits for participation. All of them had normal or corrected-to-normal visual acuity and reported no evidence of neurological compromise. The study was approved by the Institutional Review Board and informed written consent was obtained from each participant.

#### Materials

For a lexical decision task, a total of 610 high frequency nouns (no proper names, no animals) with a length of 4–9 letters were selected from the CELEX database (Baayen et al., 1995). For each word a non-word was generated by keeping the position of the first and the last letter while randomly changing the position of the middle letters, resulting in a pool of 1220 stimuli for the lexical decision task.

From these materials five different blocks were composed. A baseline block contained 40 pseudo-randomly selected trials (20 words and 20 non-words). Four experimental blocks contained 305 trials composed of 295 letter-strings from the stimulus pool and 10 additional prospective memory targets. The word “Hund” (German for “dog”) which is the most typical member of the category animal served as the prospective memory target (Hager and Hasselhorn, 1994). A specific rather than a categorical prospective memory target was used because in everyday life habitual prospective memory tasks are generally cued by specific target events (e.g., taking medication after breakfast; cf., Meacham and Leiman, 1982; Dismukes, 2008). In each block, the first prospective memory target was presented at the 30th position and the last (i.e., the 10th) was presented at the 300th position, respectively. The remaining prospective memory targets were presented at pseudo-randomized intervals of 20, 30, or 40 trials between the first and the last prospective memory target. Across experimental blocks, a total of 100 four-letter-words were randomly distributed. These were used as control items.

Stimuli were presented in uppercase and in 36-point black Arial font against a white background in the center of the screen. The letter-strings were surrounded by a black rectangle with a border width of 2 mm which remained on the screen during the whole experiment. The purpose of the rectangle was to facilitate the fixation of the center of the screen and to minimize eye-movements. The experiment was controlled by E-Prime 1.1 software (Psychology Software Tools, www.pstnet.com) running on an IBM-compatible computer with a 17” VGA monitor.

#### Procedure

After obtaining consent, the electroencephalography (EEG) recording equipment was set up and the participants were instructed for the lexical decision task. Specifically, they were informed that they would see letter-strings on the computer screen and that for each one they had to decide whether it was a word or a non-word by pressing the “B”-key with the right index finger for a word and the “N”-key with the right middle finger for a non-word. Next, EEG recording started and the baseline block was administered. Then, participants received the prospective memory task instruction. They were informed that an additional goal of the study was to investigate how well they would remember to carry out an intended activity in the future. Participants were asked to press the “H”-key on the computer keyboard with a finger of their right hand whenever the word “Hund” was presented. The test phase consisted of four experimental blocks, separated by short breaks during which participant were told to relax.

Each lexical decision trial lasted 2000 ms. First, a letter-string surrounded by a rectangle was presented for a fixed duration of 1000 ms. Then the letter-string was removed and the empty rectangle stayed for another 1000 ms resulting in a 2000 ms response window. When a participant forgot to press the “H”-key for a prospective memory target trial, a message appeared in the center of the rectangle to remind the participant of the prospective memory task. To continue, participants were instructed to press the “H”-key. This procedure was used to make sure that the task became habitual and that a large number of prospective memory target trials was available for the ERP-analysis. The whole experiment lasted ∼50 min.

#### EEG recording and analysis

The EEG was digitized (500 Hz, 0.015 to 250 Hz bandpass) and stored from 62 electrodes located according to an extended version of the International 10–20 System using a Brainproducts EEG system. Inter-electrode impedances were kept below 5 kΩ. All electrodes were recorded against Fz. Eye-movements were monitored with two additional electrooculogram (EOG) channels.

For oﬄine data analysis, first, an independent component analysis (ICA) based eye-movement correction was applied (Delorme et al., 2007). Across subjects, between one and three ICA components were considered as related to horizontal and vertical eye-movements and were thus removed. Further periods with remaining artifacts were identified and removed according to visual inspection. Electrodes F1 and F2 had to be excluded in all datasets due to technical problems. The data were filtered oﬄine with a bandpass filter from 0.5 to 20 Hz, the reference channel Fz was reinstated and the data were recomputed against average reference. Artifact-free EEG epochs were extracted from 100 ms before stimulus presentation to 1000 ms after stimulus presentation for correct responses. No pre-stimulus baseline correction was applied to avoid confounding effect of an eventual pre-stimulus CNV.

#### Identification of the prospective memory modulations

A first analysis was conducted to identify the three prospective memory components N300, parietal old/new effect, and sustained parietal positivity (West, 2005, 2008). Separate ERPs for prospective memory target trials and for four-letter control words from the ongoing task were computed and averaged across subjects. The differences between ongoing task ERPs and prospective memory target ERPs were calculated in three post-stimulus time windows derived from the literature: from 250 to 450 ms (N300), from 450 to 650 ms (parietal old/new effect), and from 650 to 850 ms (sustained parietal positivity).

All ERP comparisons were based on paired topographic analyses of variances (TANOVAs), normalized across electrodes, as a global test for topographic differences (Strik et al., 1998). TANOVAs have been shown to yield similar conclusions as previously used statistical analysis (Wirth et al., 2007), but minimize problems of redundant testing across electrodes or pre-selection of sites for testing. Differences that were significant in the TANOVA were further explored using paired *t*-maps, informing about the scalp distribution of the signal-to-noise ratio of an effect and allowing comparisons with previous studies. Furthermore, since topographic differences assessed by a TANOVA must have resulted from differences in active brain regions, significant TANOVA differences were also investigated using voxel-wise *t* tests of low resolution brain electromagnetic tomography analysis (LORETA, Pascual-Marqui et al., 1994). For source localisation, software from the Cuban Neuroscience Center, Havanna was used, employing an average brain model of the Montreal Neurological Institute (Collins et al., 1994). A forward model consisting of three spheres was used to model piecewise homogenous compartments of the brain, skull and scalp, with radios of 95, 99, and 103 mm respectively. As conductivity ratios 1, 0.0125, 1 for the brain, skull and scalp, respectively, were used (cf., Oostendorp et al., 2000; Zhang et al., 2006). A grid of 3244 points constrained to the gray matter modeled the intracerebral electrical sources. The grid has a resolution of 7, 7, and 8 mm for X, Y, Z axes, respectively. With this information the physical term (electric lead field) that relates the intra-cerebral activity to scalp electric fields was computed. Inverse solutions of the individual mean maps in the significant analysis window were computed for each condition using the LORETA method, normalized for variance across voxels, and a paired *t* test was computed in each voxel. The following contrasts were investigated:

1. Event-related potential differences between the prospective memory and the ongoing task. This is a replication of previous findings and can be considered as *prospective memory effect*.
2. Event-related potential differences between the first and second half of the experiment for prospective memory trials. As in the first half of the experiment the prospective memory task is considered as more episodic and in the second half it is considered as more habitual this difference can be considered as the episodic to habitual *transition effect*.
3. Event-related potential differences between the first and second half of the experiment for ongoing task trials. In order to control for a more general effect that is rather related to repeatedly performing the task and which is not related to the prospective memory task *per se*, this difference can be considered as a *practice effect*.

#### Covariance mapping

Additionally, a more fine-grained analysis was conducted to investigate the trajectory of the transition effect. Rather than just comparing the first and the second half of the experiment, we tested a linear model using a covariance mapping approach. Covariance mapping allows to identify scalp fields (i.e., covariance maps) that correlate linearly with an external, continuous measure (Koenig et al., 2008). In the present case, this external measure was time, and covariance maps were computed for each subject and separately using each of the artifact-free prospective memory trials (representing the transition effect) and using each of the artifact-free trials of the ongoing task (representing the practice effect). These individual covariance maps were averaged within the early (250–450 ms), middle (450–650 ms), and late (650–850 ms) time window. Topographic consistency tests were applied to test whether the individual mean covariance maps were similar across subjects, which, if significant, would indicate that across subjects, there was a common set of brain regions that showed a linear relation of activation strength with time (Koenig and Melie-García, 2010). Furthermore, to distinguish the transition effect from the practice effect in the covariance maps, these were again compared using paired TANOVAs. The comparison of the covariance maps is mathematically identical to compute differences of prospective memory and ongoing task trials at different time points and then assess the change of this difference as a function of time.

Finally, we estimated the actual trajectory of the transition effect across the 40 trials. This analysis was based on the covariance maps obtained from the prospective memory trials averaged across subjects. The fit of all valid individual single ERP trials with these mean covariance maps across subjects was computed, separately for each time point of the analysis window, and separately for each trial (excluding wrong responses and those with artifacts). These fits were then averaged both across all time points of the analysis window and across all subjects, and plotted against the trial number.

### RESULTS

#### Behavioral data

***Prospective memory task.*** Prospective memory performance was measured as proportion of correct responses to the target word. Performance was 0.91 (SE = 0.02) for the first half and 0.93 (SE = 0.01) for the second half of the experiment, respectively. A paired-samples *t* test revealed no significant difference *t*(21) = -1.1, *p* > 0.05. Mean reaction time for correct prospective memory targets was 889 ms (SE = 24) and 854 ms (SE = 22) for the first and second part of the experiment, respectively. A paired-samples *t* test revealed a significant difference, *t*(21) = 2.3, *p* < 0.05, indicating shorter reaction times for the second compared to the first part of the experiment.

***Ongoing task.*** Proportion of correct ongoing task responses was 0.953 (SE = 0.006) for the first and 0.950 (SE = 0.008) for the second half of the experiment. A paired-samples *t* test revealed no significant difference, *t*(21) = 1.1, *p* > 0.05. Mean reaction time of the ongoing task trials (correct responses) was 690 ms (SE = 15) and 683 ms (SE = 15) for the first and the second part of the experiment, respectively. A paired-samples *t* test revealed no significant difference *t*(21) = 1.4, *p* > 0.05.

To test whether performing the ongoing lexical decision task was affected by the additional requirement of the prospective memory task, the difference between lexical task performance in the ongoing task and in the baseline trials was calculated. Mean reaction time difference was -37 ms (SE = 16) and -46 ms (SE = 18) for the first and the second part of the experiment, respectively. Accuracy difference was -0.01 (SE = 0.01) both between the practice and the first part as well as between the practice and the second part of the experiment. The results of *t* tests showed no costs associated with performing the prospective memory task (all *t*s ≤ 1.5; all *p*s > 0.05).

#### Electrophysiological data

***Identification of the prospective memory modulations.*** The TANOVAs comparing prospective memory target trials and control words revealed significant effects in the early time window (250–450 ms, *p* < 0.001), in the middle time window (450–650 ms, *p* < 0.05) and in the late time window (650–850 ms, *p* < 0.001), respectively.

**FIGURE 1:**
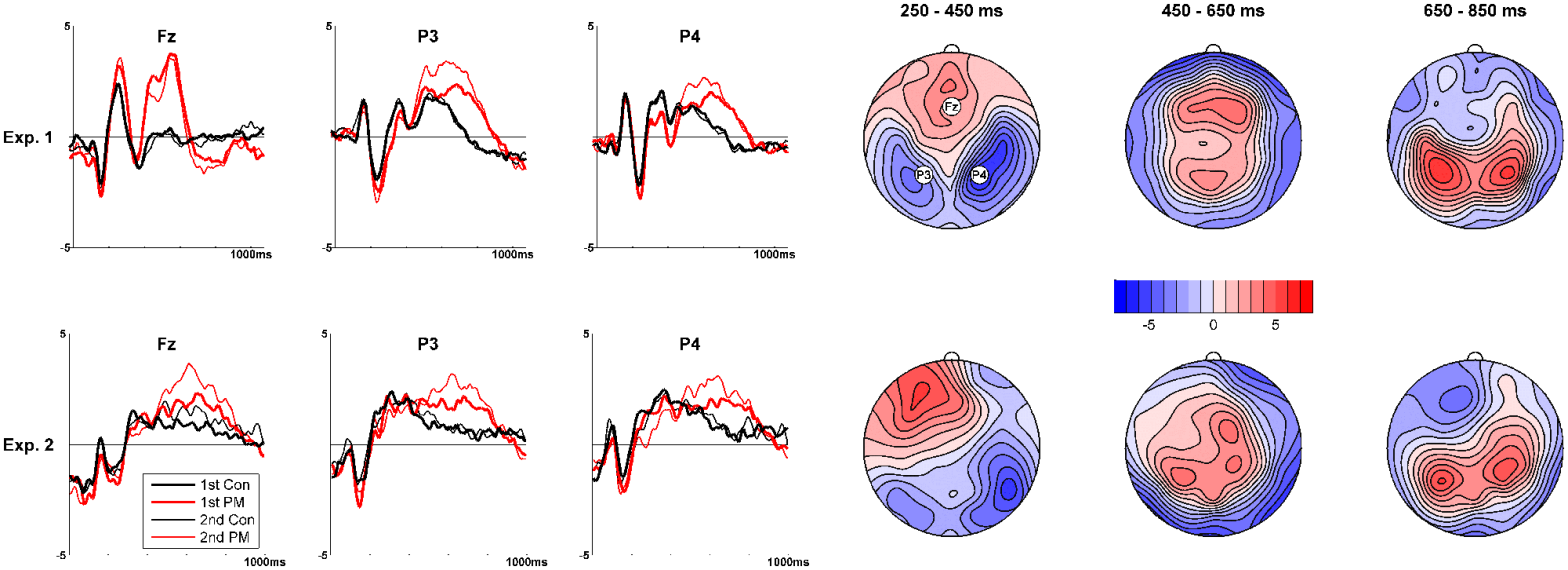
**Prospective memory components for Experiment 1 and Experiment 2.** Left: ERPs for prospective memory targets (PM; red lines) and control trials (Con; black lines) at selected electrodes, for first (1st) and second half (2nd), respectively. Right: *t*-maps comparing the ERPs from the prospective task with the ERPs from the ongoing task for the early (250–450 ms), middle (450–650 ms), and late time windows (650–850 ms), reflecting the N300, the parietal old/new effect and the late parietal positivity, respectively. The colorbar indicates *t*-values.

On top of **Figure 1** the grand-mean traces of prospective memory target trials and control words at electrodes where their differences were most pronounced are presented (left), and the *t-*maps of differences between the two conditions (right). In the early time window, the *t-*maps revealed a bilateral negativity at electrodes over occipital, parietal, and temporal regions of the scalp indicating the N300 (largest *t*-value at electrode P4, *t* = 7.7). This was accompanied by positivity at frontal electrodes (cf., West et al., 2001; West and Krompinger, 2005). For the middle time window, a positivity was found at frontal, central, and parietal regions of the scalp, indicating both a P3b and a parietal old/new effect (largest *t*-value at electrode F4, *t* = 4.4). For the late time window, a positivity was found at parietal electrodes only, indicating the sustained parietal positivity (largest *t*-value at electrode P3, *t* = 6.4). Therefore, the typical ERP modulations for prospective memory were identified which is a pre-condition for further analyses.

**FIGURE 2:**
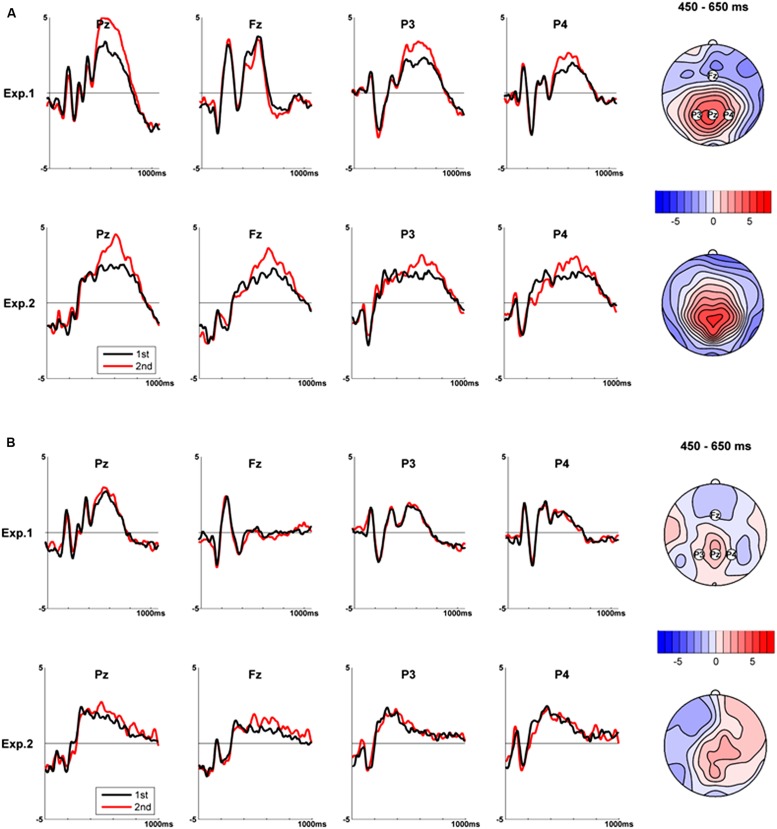
**Comparison of first versus second test halves for Experiment 1 and Experiment 2. (A)** Transition effect. **(B)** Practice effect. Left: ERPs for prospective memory targets **(A)** and control items **(B)** from the first (black lines) and second half (red lines) at selected electrodes. Right: corresponding *t*-maps in the middle time window (450–650 ms).

***Analysis of the episodic to habitual transition effect.*** The analysis of the transition effect was based on an average of 17.8 (range = 15–20) valid prospective memory target trials per subject from the first half, and 18 valid prospective memory target trials (range = 15–20) from the second half. TANOVAs comparing the first and second half ERPs in the three time windows yielded a significant difference in the middle time window (*p* < 0.001), but neither in the early nor in the late time windows (*p* = 0.23 and *p* = 0.14, respectively). **Figure 2A** (top) shows the *t*-map and selected traces of the transition effect in the significant time window. The largest differences were observed at parietal electrodes (largest *t*-value at electrode PO1, *t*-value = 5.0). The traces at the selected electrode Pz show higher amplitudes in the second half (printed in red color) compared to the first half (printed in black color) of the experiment in the middle time window. Our data therefore suggest that the transition of the prospective memory task from episodic to habitual affected either the P3b which would indicate a reallocation of processing capacity or the parietal old/new effect, which is thought to represent the retrospective component of prospective memory.

**FIGURE 3:**
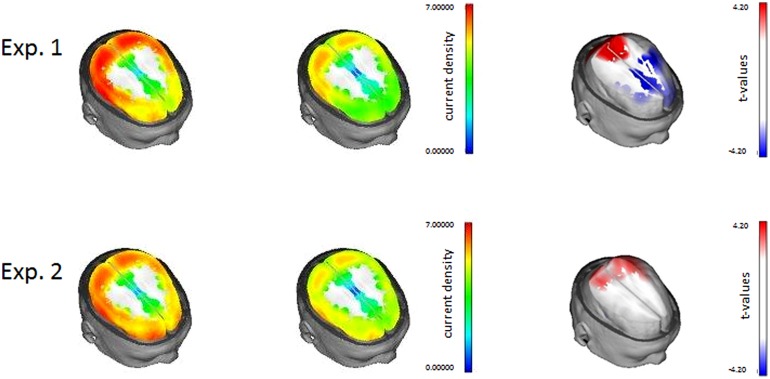
**Results from low resolution brain electromagnetic tomography analysis (LORETA) for Experiment 1 (top) and Experiment 2 (bottom).** On the left side the 1st and 2nd half data are presented, the colorbar indicates current density. On the right side the transition effect is presented; the colorbar indicates *t*-values.

Low resolution brain electromagnetic tomography analysis voxel-wise statistics are shown in **Figure 3** and indicate that the transition from episodic to habitual prospective memory was associated with an increase in activity in parieto-occipital areas and a decrease in frontal activity. Statistically, regions with significantly higher current density in the second half were identified in occipital and superior parietal brain areas and regions with significantly lower current density in the second half were spread across superior, medial and inferior frontal brain areas (*p* < 0.05).

***Analysis of the practice effect.*** The analysis of the practice effect was based on four-letter control words. From the first half of the experiment, on average 38.9 valid trials per subject (range = 36–41) were available and from the second half, 48.1 trials (range = 41–52) we available. None of the TANOVAs comparing first and second half ERPs was significant (*p* = 0.23, *p* = 0.29, and *p* = 0.95, for the early, middle, and late time windows, respectively). In **Figure 2B** (top), the shapes of the ERPs elicited by the control words from the ongoing task from the first (printed black color) and the second half (printed in red color) are presented. The two waves did not show any apparent differences. Thus, when comparing ERPs recorded in the second against the first half of the experiment, the presence of a significant effect obtained in the prospective memory trials, and the absence of an effect in the control task supports the notion that the transition effect is specific for prospective memory.

***Covariance mapping.*** For the prospective memory trials, the covariance analysis with time as linear predictor yielded covariance maps that were consistent across subjects in the middle (*p* < 0.001) and late (*p* < 0.001) time window, but not in the early time window (*p* > 0.99). These covariance maps revealed a central posterior positivity. Covariance analysis of the practice effect was significant in the early (*p* < 0.001) and in the middle time window (*p* < 0.05), but not in the late window (*p* > 0.99). To compare the differences between the covariance maps of the prospective memory and the control trials, TANOVAs were computed with normalized data. The results showed no difference in the early time window (*p*= 0.89), but the covariance maps differed significantly in the middle and the late time window (both *p* < 0.05).

**FIGURE 4:**
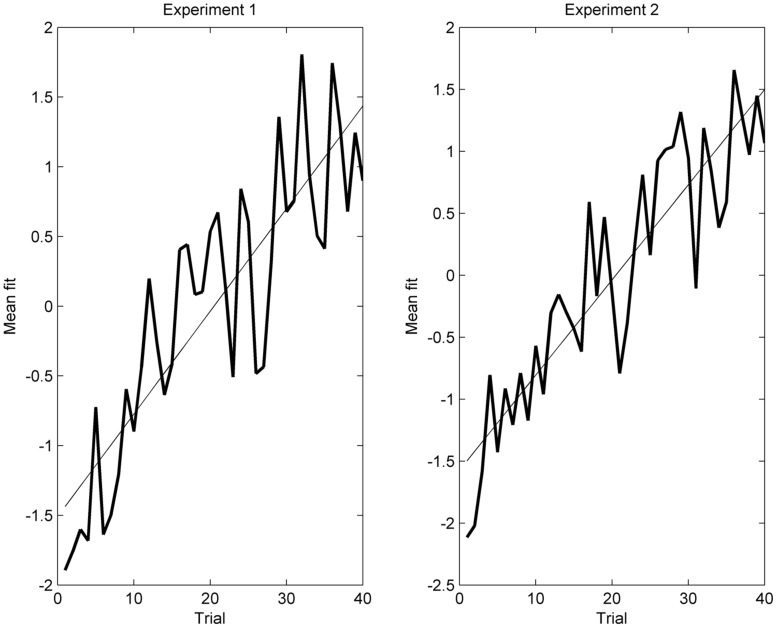
**Mean fit across all subjects of the single trial ERP (vertical axis) with the mean covariance map plotted against trial number (horizontal axis).** The more positive the value, the more the single trial ERP data resembled the obtained covariance maps. Experiment 1(left), Experiment 2 (right). Both experiments showed a rather continuous increase of the fit that was well approximated with a linear regression line.

The estimated trajectory of the transition effect across the 40 trials is shown in **Figure 4** (left side). As expected, there was an overall negative fit with the covariance maps in the trials of the first (more episodic) half of the experiment, and a positive fit in the second (more habitual) half. The fit with the covariance maps can be interpreted as an index for the transition from a more episodic to a more habitual mode of processing. It appears that this transition is rather linear. Indeed, a linear regression of the points of the trajectory explained 74% of the variance (*r* = 0.86).

### DISCUSSION

The primary goal of Experiment 1 was to test whether the neural signature changes when a prospective memory task changes from episodic to habitual. In order to accomplish this goal we first tested whether we would find the three neural components which are typically associated with a prospective memory task (i.e., the N300, the P3b, the parietal old/new effect, and the sustained parietal positivity). As expected, these components were identified in an early, middle, and late time window, respectively and therefore the precondition for testing the transition from episodic to habitual prospective memory was met. Next, we compared these components in the first and the second half of the experiment. We found a difference in the middle time window only. That is, the parietal old/new effect was stronger in the second compared to the first half of the experiment as expressed by an increased activation at centro-parietal electrodes and a decrease of activation at frontal electrodes. Using LORETA, this transition effect was localized in parieto-occipital and frontal brain regions. Specifically, when the task changed from episodic to habitual there was an increase in brain activity in parieto-occipital areas and a decrease in brain activity in frontal areas. Covariance mapping further revealed that the differences between the ERP activation patterns in the first half and the second half of the experiment for prospective memory targets compared to control words was significant mainly in the middle and the late time windows, which are both associated with parietal activations. Finally, a plot of the fit across each single ERP trial for each individual with the covariance map across all participants revealed a linear relationship, indicating that the transition from episodic to habitual is rather continuous than categorical.

The results confirm that episodic and habitual prospective memory can be differentiated on a neural level. However, the distinction is rather quantitative than qualitative, as in both halves of the experiment the three components of prospective memory were found. As the only difference was found in the middle time window, in which the P3b and the parietal old/new effect occurred, the critical difference seems to be related either to a reallocation of processing capacity or to a facilitation of retrieval processes. The first interpretation is consistent with findings from dual-task studies of the oddball paradigm which showed that with more difficult primary tasks fewer resources are available for the secondary task which is expressed in decrease of P3b amplitude (e.g., Kramer et al., 1991; Watter et al., 2001). As the resource demands for the ongoing task decrease with practice a reallocation of processing capacity to the prospective memory task in the second half of the experiment is likely. However, the findings would also be consistent with results from research in recognition memory where, similarly, an increase of the parietal old/new effect occurs with high confidence in recollection (e.g., Curran, 2004). The result of a decrease in frontal activation might be related to the fact that the more habitual the prospective memory task becomes, fewer resources must be recruited for monitoring the prospective memory targets. This interpretation would be consistent with results from functional magnetic resonance imaging (fMRI) studies showing the involvement of frontal areas when monitoring for the prospective memory targets is required (e.g., Gilbert et al., 2006; Simons et al., 2006).

On a behavioral level, the accuracy of performing the prospective memory task was high from the beginning and close to ceiling. This was intended by design in order to include as many valid EEG-trials as possible in the ERP-analyses. The results further showed that performing the prospective memory task became faster in the second compared to the first half while performance of the lexical decision task remained constant in terms of both accuracy and response times. Together the behavioral results suggest that when the prospective memory task changed from episodic to habitual, performance became faster and this was not accompanied by a cost in ongoing task performance. This result indicates that, in fact, performing the prospective memory task required less attention and its execution became more automatic (cf., Einstein et al., 1998; Dismukes, 2008). Thus, the combination of the behavioral results and also the inspection of the *t*-maps are in line with the interpretation of resource allocation and the P3b as the source of the difference between the first and the second part of the experiment.

In Experiment 1 we used one specific prospective memory target which was presented repeatedly across the experiment. In contrast, the control word condition was composed of different four-letter words that were not repeated across the experiment. Therefore, it is possible that our results have been influenced by the fact that compared to control words the prospective memory targets became more familiar across the experiment. As a consequence an alternative explanation would be that the differential effects are simply related to prospective memory target familiarity. In order to exclude this alternative explanation we designed a second experiment. In Experiment 2 we used categorical rather than specific prospective memory targets. In addition, the control items were closely matched to the prospective memory targets. Moreover, in Experiment 2, we used a non-verbal perceptual discrimination task rather than a (verbal) lexical decision task. We reasoned that if the results from Experiment 1 are in fact specific to the transition between an episodic and a habitual prospective memory task, then the same pattern of results should be found in Experiment 2, independent of the particular ongoing task and independent of the particular kind of prospective memory targets.

## EXPERIMENT 2

### METHOD

#### Participants

A total of 20 new right-handed volunteers who had not participated in Experiment 1 were recruited. One participant had to be excluded from the analyses due to evidence of neurological compromise and two participants had to be excluded due to perspiration artifacts in the EEG-data. The data of the remaining 17 participants were used (mean age = 28 years, SD = 4; 10 female). All of them had normal or corrected-to-normal visual acuity and no evidence of neurological compromise. The study was approved by the Institutional Review Board and informed written consent was obtained.

#### Materials

For a perceptual discrimination task, a total of 1240 abstract shapes were used. From the materials of Slotnick and Schacter (2004) we selected 1200 shapes, subdivided into 30 differently colored sets each with 40 different shapes. A set of white shapes was used for the prospective memory task. In order to form a control condition, the set of white shapes used for the prospective memory task was dyed with a light yellow color. Accordingly, prospective memory items and control items consisted of exactly the same shapes and differed only by their color. For each color set five identical and five non-identical shape-pairs were created. The materials were equally divided into four experimental blocks. Each block consisted of 310 different shapes in 31 different colors. A particular shape appeared twice in each block, once in an identical shape-pair and once in a non-identical shape-pair.

For practice 36 trials with 18 identical and 18 non-identical shape-pairs with six different colors were used. These were different from the shapes of the experimental blocks.

As in Experiment 1 the first and the last (10th) prospective memory targets were positioned at the 30th and the 300th trial of every experimental block. The remaining prospective memory targets were distributed between them at pseudo-randomized intervals of 20, 30, or 40 trials. To counterbalance the relative order of prospective memory and control trials, the first and the last (10th) control targets were presented as 15th and 285th trial in two of the blocks, and as 40th and 310th trial in the other two blocks, respectively, the remaining control targets were presented at pseudo-randomized intervals of 15, 20, 25, 30, 35, 40, or 45 trials. Block order was randomized for each participant.

#### Procedure

The procedure of Experiment 2 was similar to Experiment 1. For the perceptual discrimination task participants were informed that they will see a pair of shapes on the computer screen and that they have to decide whether the two shapes are identical or not by pressing the “B” or the “N”-key with the index finger and the middle finger of the right hand. For the prospective memory task, participants were instructed to press the “M”-key with the ring finger of the right hand whenever a white shape-pair was presented.

Each perceptual discrimination trial lasted 2000 ms. Stimuli were presented in pairs, side by side horizontally, against a black background, in the center of the screen. A gray fixation-cross was presented between the two shapes and the shape-pairs were surrounded by a gray rectangle. A colored shape-pair was presented for 1000 ms, then the shape-pair was removed and the fixation-cross and the rectangle stayed for another 1000 ms, resulting in a 2000 ms response window. The whole experiment lasted ∼50 min.

#### EEG recording and analysis

The EEG was digitized (500 Hz, 0.015 to 250 Hz bandpass) and stored from 64 electrodes located according to an extended version of the international 10–20 system using an Easycap EEG system. Inter-electrode impedances were kept below 5 kΩ. All electrodes were recorded against Fz. Eye-movements were monitored with two additional EOG channels.

First, the sampling rate was reduced to 200 Hz. Across subjects, between 1 and 3 ICA components (Delorme et al., 2007) were recognized as eye-movement related and oﬄine removed from the data. Based on visual inspection further periods with artifacts were removed from further analysis. The data were filtered oﬄine using a bandpass filter from 0.5 to 20 Hz and recomputed against average reference. Artifact-free EEG epochs were extracted from stimulus presentation to 1000 ms after stimulus presentation for correct responses.

In order to identify the prospective modulations the same analyses were conducted as described in Experiment 1. The early time window lasted from 250 to 450 ms after stimulus (representing the N300), the middle time window lasted from 450 to 650 ms after stimulus-onset and the late time window lasted from 650 to 850 ms (representing the P3b, the parietal old/new effect, and the sustained parietal positivity). Further, the same analyses as in Experiment 1 were conducted with the same time windows defined above to replicate the episodic to habitual prospective memory transition effect. For a better understanding, again covariance analyses and LORETA were used.

### RESULTS

#### Behavioral data

***Prospective memory task.*** Prospective memory performance was measured as proportion of correct responses to the white target shapes. Performance was 0.75 (SE = 0.04) for the first half and 0.93 (SE = 0.03) for the second half of the experiment, respectively. A paired-samples *t* test revealed a significant difference, *t*(16) = -5.97, *p* < 0.001. Mean reaction time for correct prospective memory targets was 848 ms (SE = 24) for the first half and 834 ms (SE = 21) for the second half of the experiment. A paired-samples *t* test revealed no significant difference, *t*(16) = 0.92, *p* > 0.05.

***Ongoing task.*** Proportion of correct ongoing task responses was 0.85 (SE = 0.03) for the first half and 0.88 (SE = 0.03) for the second half of the experiment, respectively. A paired-samples *t* test revealed no significant difference, *t*(16) = -1.38, *p* > 0.05. Mean reaction time of correct ongoing task responses was 993 ms (SE = 37) for the first half and 956 ms (SE = 40) for the second half, respectively. A paired-samples *t* test revealed no significant difference, *t*(16) = 2.11, *p* > 0.05.

To test whether performing the ongoing perceptual discrimination task was affected by the additional requirement of the prospective memory task, the difference between performance in the ongoing task and the baseline trials was calculated. Mean reaction time difference was 22 ms (SE = 37) and -20 ms (SE = 37) for the first and the second half, respectively. Accuracy difference was 0.01 (SE = 0.01) between baseline and first half and 0.02 (SE = 0.02) between baseline and second half. The results of *t* tests showed no cost across the experiment (all *t*s ≤ 1.5; all *p*s > 0.05).

#### Electrophysiological data

***Identification of the prospective memory modulation.*** The comparison of prospective memory target trials and control shapes using TANOVAs and *t*-maps revealed significances in the early time window (250–450 ms), *p* < 0.001 (largest *t*-value at electrode P8: *t* = 6.6), in the second time window (450–650 ms), *p* < 0.001 (largest *t*-value at electrode FC2: *t* = 4.9), and in the third time window (650–850 ms), *p* < 0.005 (largest *t*-value at electrode P3: *t* = 5.8). *t*-maps (**Figure 1**, bottom) confirmed that the prospective memory components had a similar topography as Experiment 1; these topographies corresponded largely to those interpreted as N300, P3b, parietal old/new effect, and the sustained parietal positivity.

***Analysis of the episodic to habitual transition effect.*** For episodic prospective memory ERPs, the mean of artifact free valid trials per subject was 15.18 (range = 6–19), for individual habitual prospective memory ERPs, it was 18.35 (range = 10–20). As in Experiment 1, TANOVAs revealed a significant ERP difference of more episodic compared to more habitual trials in the middle time window, *p* < 0.005 (largest *t*-value at electrode Pz: *t* = 6.0), but not in the early or late time windows (*p*s > 0.05). The traces of the two conditions at Pz, as well as *t*-maps computed from the middle time window are shown in **Figure 2A** (bottom). This pattern is very similar to that of Experiment 1.

The results from LORETA source localisation are presented in **Figure 3** (bottom). They suggest that prospective memory ERPs of the second part compared to the first part of the experiment are associated with increased activity in the parietal lobes. This also replicates the findings of Experiment 1. Further, increased activity was also found in both occipital lobes. Brain regions with significantly higher current density in prospective memory ERPs of second compared to first half of the experiment were the precuneus, cuneus, occipital pol, superior occipital gyrus, superior parietal lobus, and inferior parietal lobus. In contrast to Experiment 1, however, ERPs of the second half of the experiment did not show a decrease in current density in the frontal lobes.

***Analysis of the practice effect.*** For computing ERPs of control stimuli, the average number of trials was 15.53, (range = 8–20) for the first half, and 16.70 (range = 9–20) for the second half of the experiment. None of the TANOVAs revealed a significant difference for the practice effect (first time window: *p* = 0.47, second time window: *p* = 0.54, and third time window: *p* = 0.33). These results are presented in **Figure 2B** (bottom).

***Covariance mapping.*** For the prospective memory trials, covariance maps were consistent across subjects in all three time windows, all *p*s < 0.001. As in Experiment 1, the covariance maps revealed positivity over frontal, central and parietal regions and negativity over frontal and temporal regions in the middle and late time window. For the control shapes, there was no evidence for consistent covariance maps across subjects in the early and late time windows (*p* = 0.58 and *p* = 0.59, respectively). However, there was a consistent topography in the middle time window (*p* < 0.001). Comparisons of covariance maps of the prospective memory trials and the control figures in the three time windows showed no significant differences in the early and late time windows (with *p*= 0.26 and *p* = 0.62, respectively), but they differed in the middle time window (*p* = 0.047).

As in Experiment 1, the estimated trajectory of the transition effect across trials indicated that the change of the covariance maps of prospective trials followed a linear gradient across the 40 trials (see **Figure 4**, on the right). Similar to Experiment 1, a regression analysis was calculated. The linear trajectory explained 81% of the variance (*r* = 0.89).

### DISCUSSION

The primary goal of Experiment 2 was to test whether the neural signature changes observed in Experiment 1 can be generalized to a non-verbal ongoing task and with categorical prospective memory intention. As in Experiment 1, we first tested the presence of the three neural components which are typically associated with a prospective memory task (i.e., the N300, the P3b, the parietal old/new effect, and the sustained parietal positivity). These components were found and therefore the precondition for testing the transition from episodic to habitual prospective memory was met. When we tested these components for changes from the first to the second half of the experiment, we found a difference in the middle time window only as in Experiment 1. This was expressed by an increased activation at centro-parietal electrodes and a decrease of activation at frontal electrodes. Using LORETA, this transition effect was accompanied by an increase in brain activity in parieto-occipital areas. In Experiment 2, there was no activation difference in frontal regions when comparing the first and second half of the experiment. The differences in stimulus material and ongoing task demands may be responsible for this result.

However, as in Experiment 1, covariance mapping also revealed differences between the ERP activation patterns in the first half and the second half of the experiment in the middle time window. A plot of the fit indices across each single ERP trial for each individual with the covariance map across all participants revealed a similar linear relationship as in Experiment 1, further supporting the assumption that on a neural level, the transition from episodic to habitual follows a continuous linear function.

On a behavioral level, prospective memory performance was lower in the first part of the experiment and increased with routine in the second, more habitual part of the experiment. As a consequence fewer data-points were available for calculating ERPs. This may have been one source for the lack of finding a reduction in frontal activations compared to Experiment 1. No accuracy differences were found for the ongoing task. Moreover, no differences were found in the reaction times, neither for the prospective memory task nor for the ongoing task. As in Experiment 1 no performance costs were associated with adding a prospective memory task, indicating the automatic nature of habitual prospective memory. To summarize, Experiment 2 replicated the main results of Experiment 1, in particular the transition effect as based on differences in parietal activations in the middle time window (between 450 and 650 ms) and thus indicates that these are independent from the particular ongoing task and the particular kind of prospective memory targets. Moreover, together the present experiments also show the generality of the transition effect across different degrees of processing overlap between the ongoing task and the prospective memory task (Meier and Graf, 2000).

## GENERAL DISCUSSION

This is the first study that addressed the transition from episodic to habitual prospective memory. In two separate experiments with different ongoing tasks and different kinds of intention specificity, we showed that with routine, the ERP-component in the time window between 450 and 650 ms post-stimulus became consistently larger. This result indicates that compared to episodic prospective memory, in habitual prospective memory resource allocation changes and intention retrieval is probably facilitated. The results confirm that episodic and habitual prospective memory can be differentiated on a neural level.

Moreover, the results indicate that the changes are rather quantitative than qualitative. This is reflected in the fact that the predicted ERP components that are typically involved in the realization of delayed intentions were present for earlier and later trials of both experiments. The N300 which is associated with prospective memory target detection was not changed when a task became more habitual. This indicates that target detection is a robust process that alerts the cognitive system that a significant event has occurred. The invariance of the N300 to habitualization is in line with result from West et al. (2003) who found a similar N300 for prospective memory targets and for prospective memory lures that were perceptually distinct. In contrast, West et al. (2006) found that the amplitude for prospective memory targets that were embedded in a 1-back task was reduced compared to when they were embedded in a 2- or 3-back task, suggesting that the neural correlates of cue detection were sensitive to the availability of attentional resources. According to this latter finding it may be surprising that with the increasing availability of processing resources associated with a task becoming habitual the N300 remained constant. However, it seems that the amount of resource changes was not sufficient to affect the N300 as it was when West et al. compared 2- and 3-back trials.

The most important result of the present study is the ERP difference that was consistently found in the middle time window, in which the P3b and the parietal old/new effect occurred. Thus, as a task becomes habitual a reallocation of processing capacity, a facilitation of retrieval processes, or a combination thereof seems to occur. It is possible that the transition effect reflects memory retrieval in the earlier trials and a reallocation of processing capacity in the later trials once ongoing task processing is proceduralized and the representation of the prospective memory target has stabilized. This interpretation is consistent with findings from dual-task studies of the oddball paradigm which showed that with more difficult primary tasks fewer resources are available for the secondary task which is expressed in decrease of P3b amplitude (e.g., Kok, 2001; Watter et al., 2001). By the same logic, with increasing practice the ongoing task gets easier, thus freeing resources for the prospective memory task as expressed by an increase of P3b amplitude. It is also consistent with results from recognition memory in which high confidence in recollection is associated with an increased parietal old/new effect and with the results of a recent prospective memory study that showed enhanced prospective positivity for easier compared to more difficult prospective memory targets (Cona et al., 2013). Notably the present study was not designed to distinguish between these two possibilities. We were motivated by a more modest goal, namely to test whether we would find any differences in the neural signature of the transition between episodic and habitual prospective memory. Future studies are necessary to test the relative contribution of the P3b and the parietal old/new effect for this transition.

Finally, we also found a robust sustained parietal positivity which was not changed in the course of the experiment. Although the functional role of this late component is not clear yet it seems to be related to post-retrieval processes or as suggested more recently by processes related to task-set reconfiguration (Bisiacchi et al., 2009; West, 2011). This idea is consistent with the result that the sustained parietal positivity was larger for prospective memory targets than for prospective memory lures in a study by West et al. (2001). In the context of the present study it is reasonable to assume that these processes and task requirements remain stable.

On a behavioral level, the accuracy of performing the prospective memory task was high from the beginning and close to ceiling. This was intended by design in order to include as many valid trials for the ERP-analyses. In Experiment 1, the results further showed that performing the prospective memory task became faster in the second compared to the first half while performance of the lexical decision task remained constant. In Experiment 2, prospective memory performance increased while ongoing task performance remained constant. Together the behavioral results suggest that when the prospective memory task changed from episodic to habitual, performance became faster and this was not accompanied by a cost in ongoing task performance. This result indicates that, in fact, performing the prospective memory task required less attention and its execution became more automatic (cf., Einstein et al., 1998; Dismukes, 2008). In line with this interpretation, responding to prospective memory targets became statistically (Experiment 1) and numerically (Experiment 2) faster, however, due to the bivalent nature of prospective memory targets, performance did not reach the level of the ongoing task (cf., Meier and Rey-Mermet, 2012).

On a neuroanatomical level, further analyses involving source localisation revealed that the neural changes associated with the transition effect are related to an increase in activity in parietal brain areas and, at least in Experiment 1, it was also related to a decrease in frontal brain activity. Frontal cortex activation has generally been discussed as reflecting “retrieval effort” (Schacter et al., 1996) and as being involved in strategic and intentional retrieval of stored representations (Fletcher and Henson, 2001). For example, Simons et al. (2006) found higher activation in frontal regions, specifically in lateral BA 10 (and deactivation in medial BA 10) associated with cue identification and also intention retrieval. These effects were more pronounced when the prospective memory task required higher demands on the retrieval of the intention. Gilbert et al. (2006) found that reaction times to tasks that had provoked lateral BA 10 activations were slower than reaction times in their control tasks. Lateral BA 10 is presumably activated whenever additional attention resources are spent to an external stimulus and when resources are invested to handle this stimulus (Burgess et al., 2008). In the present study episodic to habitual prospective memory transition was accompanied by a frontal deactivation on the neuronal level and shorter reaction time on the behavioral level indicating that retrieval was more automatic and less attention resources had to be spent in more habitual compared to more episodic prospective memory task trials.

Parietal cortex activation is often involved in episodic memory retrieval (Cabeza and Nyberg, 2000; Wagner et al., 2005). According to the mnemonic-accumulator hypothesis (Wagner et al., 2005), memory strength is expressed by activity in the parietal cortex which is assumed to temporally integrate memory-strength signal. Thus, the higher parietal activation identified in habitual prospective memory with ERPs and LORETA is consistent with fMRI findings of higher confidence in the recollection of the intended action.

Overall, the pattern of decreased frontal activation and increased parieto-occipital activation that accompanies the transition from episodic to habitual prospective memory is compatible with the multi-process framework of prospective memory (cf., McDaniel and Einstein, 2000; Meier et al., 2006, 2011). According to this framework, one route toward successful prospective memory is via reflexive associative processes. It is assumed that retrieval cues interact with memory traces previously associated with the cues and deliver the intention reflexively to awareness (McDaniel et al., 2004). After repeated performance of the prospective memory task memory traces may be stronger and the association between cue and intention more pronounced supporting reflexive associative processes.

## Conflict of Interest Statement

The authors declare that the research was conducted in the absence of any commercial or financial relationships that could be construed as a potential conflict of interest.
